# Developing a digital communication training tool on information-provision in oncology: uncovering learning needs and training preferences

**DOI:** 10.1186/s12909-018-1308-x

**Published:** 2018-09-24

**Authors:** Sebastiaan M. Stuij, Nanon H. M. Labrie, Sandra van Dulmen, Marie José Kersten, Noor Christoph, Robert L. Hulsman, Ellen Smets, on behalf of the INSTRUCT project group, Stans Drossaert, Hanneke de Haes, Arwen Pieterse, Julia van Weert

**Affiliations:** 1 Department of Medical Psychology, Academic Medical Centre, Amsterdam, The Netherlands; 2 NIVEL (Netherlands institute for health services research), Department of Primary and Community Care, Utrecht, The Netherlands; 3 Radboud University Medical Center, Nijmegen, The Netherlands; 4 Faculty of Health and Social Sciences, University College of Southeast Norway, Drammen, Norway; 5 Department of Haematology, Academic Medical Center, Amsterdam, The Netherlands; 6 Center for Evicence Based Education, Academic Medical Centre, Amsterdam, The Netherlands

**Keywords:** E-learning, Doctor-patient communication, Focus group, Oncology, User-centred design

## Abstract

**Background:**

Adequate information-provision forms a crucial component of optimal cancer care. However, information-provision is particularly challenging in an oncology setting. It is therefore imperative to help oncological health care practitioners (HCP) optimise their information-giving skills. New forms of online education, i.e. e-learning, enable safe and time and location independent ways of learning, enhancing access to continuous learning for HCP.

As part of a user-centred approach to developing an e-learning to improve information-giving skills, this study aims to: 1) uncover the learning needs of oncological healthcare providers related to information- provision, and 2) explore their training preferences in the context of clinical practice.

**Methods:**

Focus groups and interviews were organised with oncological HCP (medical specialists and clinical nurse specialists) addressing participants’ learning needs concerning information- provision and their training preferences with respect to a new digital training tool on this issue. All sessions were audiorecorded and transcribed verbatim. Using an inductive approach, transcripts were independently coded by three researchers and discussed to reach consensus. Main themes were summarised and discussed.

**Results:**

Four focus group sessions (total *n* = 13) and three interviews were conducted. The first theme concerned the patient outcomes HCP try to achieve with their information. We found HCP to mainly strive to promote patients’ understanding of information. The second theme concerned HCP reported strategies and challenges when trying to inform their patients. These entailed tailoring of information to patient characteristics, structuring of information, and dealing with patients’ emotions. Regarding HCP training preferences, an e-learning should be neatly connected to clinical practice. Moreover, participants desired a digital training to allow for feedback on their own (videotaped) information-giving skills from peers, communication experts, and/or patients; to monitor their progress and to tailored the training to individual learning needs.

**Conclusions:**

An e-learning for improvement of information-giving skills of oncological HCP should be aimed at the transfer of skills to clinical practice, rather than at enhancing knowledge. Moreover, an e-learning is probably most effective when the facilitates individual learning needs, supports feedback on competence level and improvement, and allows input from significant others (experts, peers, or patients).

## Background

While it has consistently been shown that cancer patients value information, their information needs are still often not met [1–3]. These unmet information needs mainly include the discussion of possible side-effects of treatment, prevention or reduction of side-effects at home, and treatment that can reduce side-effects [4, 5]. Adapting information to patient needs helps them in decision making and preparing for the consequences of treatment; increases their adherence; and improves trust and satisfaction [6–9]. Information can also reduce patients’ distress and ultimately promote their recovery [10–12]. Adequate information provision thus forms a crucial component of optimal cancer care. However, information-provision in an oncology setting is particularly challenging, not only because of the emotional impact of a potentially life threatening disease, but also because of the high information density and complexity and the availability of multiple treatment options. Inadequate information-provision may result from time constraints during consultations [13], health care professionals’ (HCP) unawareness of a patient’s specific information needs [14–16], the complexity of discussed information, and the usage of medical jargon [17]. Additionally, HCP may withhold information, because they worry that too much information will increase patient anxiety [18]. On top of this, patients forget on average 50% of the information provided during consultations [19]. Given all these limiting factors, it is imperative to help oncological HCP improve their information-giving skills.

Available communication training for HCP often relies on a ‘classical’ approach in which participants have to attend face-to-face tutoring sessions, which are time-consuming, inconvenient to schedule, and costly [20, 21]. In recent years, new forms of online education, i.e., e-learning, are available using online interactive and multi-media technologies, even on mobile devices. E-learning enables new time and location independent ways of learning, enhancing access to continuous learning for HCP. We interpret e-learning in its broadest sense as ‘instructions delivered on a digital device that is intended to support learning’ [22], i.e., any digital training tool ranging from web based e-learning modules to simulation training in a virtual world. E-learning is most often used for knowledge transfer, but it can also be used for skills-based training [20]. Furthermore, e-learning offers learners the opportunity to practice these skills in a safe environment, without direct consequences for patients and without peer-pressure, in their own preferred time and learning environment. E-learning also allows for immediate feedback and personalized content to meet the individual needs of the learner. Systematic reviews on the effectiveness of e-learning on HCPs’ behaviour and patient outcomes suggest that e-learning can be at least as effective as traditional learning approaches, and superior to no training at all [23, 24].

To maximize the chances of acceptance and usage in practice, it is generally recommended to include ‘end-users’ in an iterative and cooperative fashion during each stage of the development process of an e-learning [25]. This results in applications based on users’ wishes and preferences [26]. Moreover, as with any training design process, end users or potential trainees can be helpful for establishing the specific learning needs and training gap. Pollak et al. [21] also advocate this approach for the medical context; they state that “... we need to engage clinicians in the same way we ask them to engage patients – strategically and with empathy…”. We plan to develop a digital training tool that may help oncological HCP communicate treatment information more effectively to their patients. To the best of our knowledge, based on a preliminary review of the literature, no such e-learning has been developed yet. We found 13 studies which described and evaluated digital communication skills training programs for HCP, yet none of these involved oncological HCP; they were targeted at (medical) students [20, 27–33], resident physicians/doctors-in-training [34, 35], general practitioners [34, 36–38], or nurse practitioners [37]. Moreover, they were aimed at improving participants’ general communication skills, rather than the specific skill of information-provision (unpublished thesis).

For the process of developing our tool, we chose to use the CeHRes Roadmap [39], which is a user-centred and holistic framework for developing digital health related applications and interventions. Sequential (iterative) steps include contextual inquiry, value specification, design, operationalization and summative evaluation. This study deals with the first step in the developmental process of the training: the contextual inquiry. More specifically, the aim of this study is: 1) to uncover the learning needs of healthcare providers in an oncology setting related to providing information skills, and 2) to explore their training preferences in the context of clinical practice.

## Methods

### Participants and procedure

Since information-provision in oncology is a multidisciplinary effort requiring the aligned effort of various HCP, we interviewed medical specialists (haematologists and radiotherapists) and clinical nurse specialists involved in the care for cancer patients. To maximise possible variation in needs and preferences, we aimed to include participants varying in age, gender, and clinical experience; from academic as well as general hospitals. Furthermore, we strived for variation in experience with modern technology, e-learning, and degree of enthusiasm towards e-learning. Participants were recruited through a snowball approach using e-mail, phone, and face-to-face meetings at, for example, oncology related congresses. The study was performed in accordance with the Declaration of Helsinki; The Medical Ethics Committee of the Academic Medical Center waived the requirement for ethical approval (W16_054#16.069).

A focus group was scheduled per hospital location and per specialism. Up to 5 people were scheduled per focus group session of approximately 1.5 h. Interviews of approximately 1 h were scheduled separately with HCP who could not attend the focus group session. Participants signed an informed consent form and filled out a questionnaire concerning demographic characteristics (age, discipline, department, and years of professional work experience). Furthermore study specific items addressing views towards their own communication skills (1 item), their wish to improve these skills (2 items), and their technical competence (1 item) were included (see Table 1). Focus group sessions and interviews were digitally audiorecorded. The focus groups were accompanied by a facilitator (NL) who assisted before and during the session with the recording and time-management and who noted relevant observations. During focus group sessions and interviews, two general domains were discussed: 1) the learning needs of participants’ concerning information-provision, and 2) their training preferences with respect to a new digital training tool on this issue. A topic guide with open-ended questions was used to guide the discussion in a semi-structured fashion (see Appendix A for examples of the questions). Questions in the ‘learning needs’ domain concerned participants’ goals when informing patients, challenges faced when delivering information, and reflection on factors that could influence information-provision. Questions in the ‘training preferences’ domain concerned participants’ experience and attitude towards face-to-face and digital training in general and more specifically related to communication training. Finally, perceived barriers and facilitators for a new digital training were questioned. The topic guide was created by two researchers (SS, NL) and was reviewed by members of a special interest group committed to this project (INSTRUCT project group), encompassing experts with a background in medical communication, education, and oncological care. Subsequently, minor adaptions were made. Furthermore, minor adjustments were made after the first focus group session, resulting in similar but clearer questions.

**Table 1:** Participant characteristics (n = 16)

Gender	10 female; 6 male
Age	Median: 38 years (interquartile range: 8 years)
Specialty	
- haematology	8
- radiotherapy	2
- nursing	6
Medical centre	
- academic (4)	13
- peripheral (2)	3
Professional work experience	
- limited experienced (< 5 years)	3
- moderate (5–10 years of experience)	4
- high (> 10 years of experience).	9
Participants’ response to attitude statements^a^:	Median value (interquartile range)
- I am able to effectively inform my patients about treatment	8.0 (IQR = 0.3)
- I want to improve my skills regarding effective information- provision about treatment	7.5 (IQR = 2.0)
- I regard myself proficient with (new) technology	7.0 (IQR = 1.0)
- I want to enroll in a digital training concerning effective information-provision about treatment	8.0 (IQR = 1.5)

^a^Answers on a scale of 1 (totally disagree) to 10 (totally agree)

### Data analysis

Audiorecordings of the focus groups and interviews were transcribed verbatim by an external professional transcriber. Thematic content analysis was performed in several phases. First an initial codebook was constructed collectively by three researchers (SS, NL, ES) using the discussed topic guide. The MaxQDA software package (version 12) was used for coding purposes. In the codebook, a distinction was made between learning-needs and training-preferences and inductively their corresponding themes and subthemes. Transcriptions were then independently analysed and discussed by three researchers (SS, NL, ES) until consensus was reached. Themes and subthemes were reviewed and refined until researchers agreed that these reflected the essence of the complete dataset. A summary of the main findings was then constructed (SS) and checked (ES), including major themes, noteworthy exceptions, and relevant quotes.

## Results

### Participant characteristics

Four focus group sessions, with respectively 5, 3, 3, and 2 participants per session (*n* = 13), and three interviews were conducted, from December 2016 to March 2017. In total 16 HCP were included; 8 haematologists, 2 radiotherapists, and 6 clinical nurse specialists from 4 academic medical centres and 2 peripheral medical centres across the Netherlands. Four participants were in training. For further details, see Table I. Participants’ generally responded positively to the attitude statements (see Table 1).

### Effective information-provision: learning needs

Two main themes emerged with respect to participants’ learning needs. The first uncovered theme concerned patient outcomes HCP try to achieve with their information. The second theme encompasses strategies used by HCP and associated challenges when trying to inform their patients.

#### Patient outcomes strived for with information

In general, HCP wanted their patients to feel at ease during consultations; to be satisfied with the consultation; to understand what is being said during consultations and to have a clear understanding of what to expect. Furthermore HCP wanted their patients to understand and adhere to the treatment plan.

*“…my goal for the consultation is that patients understand their disease in their own wording, what the natural course of the disease is, which treatment will take place and what this treatment entails...” (Quote from highly experienced medical specialist).*

More specifically, HCP mentioned that they want to build a solid basic understanding in the first consultation, after which they can zoom in on more complex information in future consultations. In these first consultations the goal of the HCP was to get the patient to start treatment as soon as possible by discussing the diagnosis and treatment options in broad outlines. Furthermore the implications of the advised treatment are discussed including symptoms. Finally some HCP brought up that they aim to provide patients with a realistic sense of their condition, which entails the communication of the positive and negative aspects of their disease and prognosis in an appropriate ratio. According to a vast majority of clinical nurse specialists, in line with their task description, they generally discuss the treatment and side-effects more thoroughly with the patient as compared to the medical specialists. They expressed that they want their patients to understand which chemotherapy drugs they have to take, which drug causes which side-effect, what they can do to alleviate certain side-effects, and finally how and when they should reach out for help.

#### Strategies and challenges

The strategies and challenges mentioned by HCP when informing patients can be subdivided in provider-related, and system-related.

*Provider-related strategies and challenges* (see Table 2) comprise three issues: tailoring, structuring, and dealing with emotions. The issue of *tailoring* is aimed at getting to know the patient in order to tailor information to their specific situation and needs. Generally, HCP tried to get an impression of features of the patient they consider relevant, such as education level, personality, and information needs, by getting the patient to start talking as soon as possible. Another voiced tactic was to directly ask the patient about their information needs; does a patient want to hear an outline of the information or more details? This strategy was mentioned mostly by clinical nurse specialists, rather than medical specialists. HCP sometimes mentioned that they ask questions during follow-up consultations to test if important information has been remembered. The ‘teach-back’ technique, by which the patient is asked to tell what they just have been told, was sometimes mentioned. However HCP experienced this technique as patronizing towards the patient. Some HCP ask patients whether they are able to manage and understand the provided information.

*“…through the years I have learned to get the patient to start talking as fast as possible. I ask them about their expectations for the consultation and their understanding of their situation up to now. Because I want to understand the frame of mind, intelligence level, information need of the other, the interlocutor, because that is where you are going to start connecting…” (Quote from highly experienced medical specialist).*

**Table 2:** Oncological healthcare providers’ strategies and challenges relevant for information-provision

Provider related strategies and challenges		
Issue	Strategies mentioned as being applied	Challenges
Tailoring	▪ getting the patient to start talking as soon as possible, to get impression ▪ actively ask for patient needs ▪ ask for recall of previous information ▪ ask for understanding	▪ assess patients’ personality ▪ diversity (age, language, IQ, education level) ▪ prioritizing in view of time-constraints ▪ prevent jargon
Structuring	▪ provide outline, before details ▪ repeating information ▪ agenda setting ▪ summarizing	▪ prioritizing in view of time-constraints ▪ agenda setting ▪ balance pos/neg information ▪ incorporate patient questions ▪ which / how much information
Dealing with emotions	▪ acknowledge emotions ▪ asking what patient can handle emotionally ▪ showing empathy ▪ non-verbal expression ▪ social talk	▪ recognizing anxiety ▪ specific emotions, e.g., distrust, anger, dissatisfaction
System-related strategies and challenges		
	Strategies mentioned as being applied	Challenges
	▪ shared protocol for information provision ▪ written / visual / online information ▪ checklists	▪ time constraints ▪ lack of supervision / peer-review

The most important challenge for *tailoring* mentioned by HCP was the perceived high variability in personalities of patients and difficulties in assessing the ‘type’ of patient in a constrained amount of time in order to tailor their information-giving. This was mainly voiced by medical specialists as opposed to clinical nurse specialists. The following patient characteristics were mentioned as further complicating information -giving: cultural or religious beliefs, older age, non-native speaker, and low education level. Moreover, especially medical specialists, found it challenging to uncover questions patients might have, to subsequently prioritize these, and to convert medical jargon into laymen’s terms. Furthermore, most medical specialists found it difficult to fulfil individual patients’ need to know what lies ahead, because what they can communicate is generally based on the average patient. Finally, medical specialists found it challenging to take the time to discuss all options in detail, while they want to start the patient on their advised treatment as quickly as possible.

*“… for me the most challenging aspect is to determine how much information to give in a certain amount of time. Which furthermore differs from person to person. I find it difficult to estimate which patient is in need of detailed information and which patient prefers a more stepwise approach…” (Quote from medical specialist with limited experience).*

When asked about concrete learning needs with respect to *tailoring*, HCP voiced a wish to learn how to better uncover the information needs of their patients. Furthermore, they would like to receive feedback from their patients to gain a first-hand insight in their information needs. Finally, some HCP mentioned that they would like to receive scientific evidence regarding the (psychological) mechanisms relevant for tailored information-provision.

The second issue concerns *structuring* strategies, either during the consultations or over sequential consultations. Most HCP mentioned that during a consultation they often start with an outline of the information before diving into details. Repeating information was expressed as a frequently used tactic. Some HCP voiced to start their consultation by announcing the goal, applying agenda-setting, and to end with a summary. Other HCP mentioned to schedule a second consultation relatively quickly after the first consultation and to start the second consultation by repeating important information. With respect to *structuring*, HCP found it most challenging to structure and prioritize the vast amount of information within the constrained time of the consultation. They perceived the amount and complexity of information as often being too overwhelming, which hampers further information processing. Furthermore HCP found it challenging to start consultations by giving patients an overview of their situation before providing detailed information. In addition they reported problems with communicating the positive and negative aspects of the patients’ situation in an appropriate and realistic proportion. Another frequently mentioned challenge was that patients’ questions can interrupt the providers’ agenda and goal for the consultation. Finally, HCP found it difficult to determine what and how much information should be told in each separate consultation.

When asked about concrete learning needs with respect to *structuring*, HCP frequently expressed that they would like to learn how to effectively manage and prioritize information during a consultation or over consecutive consultations.

*“…which is a lot of information, really a lot. Sometimes I find it difficult to compress al this information in the first consultation with a new patient. You probably shouldn’t do this, despite wanting to give the patient an overview of his situation…” (Quote from medical specialist with limited experience).*

The third issue entails strategies for *dealing with patients’ emotions*. Most HCP mentioned the importance of acknowledging patients’ emotions during consultations because emotions impair effective information provision. A reported tactic by some HCP was to ask patients directly what they can handle emotionally. A few medical specialists brought up that they want to discuss feelings of fear before discussing treatment options and some prefer to tell good news ‘in the door opening’ in order to make patients feel at ease immediately. Being emphatic was often mentioned as an important characteristic of HCP during consultations, but no concrete strategies were mentioned. A single provider stressed the importance of non-verbal communication for conveying empathy. Some HCP mentioned the relevance of humour and social talk to break the ice and let the patient feel more at ease.

*“…when the emotions subside, after addressing them, sometimes people really have to cry for a while. There should be room for the patient to show these emotions. It’s best to get this emotional load to subside…” (Quote from highly experienced medical specialist).*

Most HCP found it challenging to deal with angry, distrustful, and dissatisfied patients. Finally, some HCP expressed difficulties with recognizing a patient’s anxiety. This is something they feel they are not trained to recognize and which especially medical specialists do not have enough time for to address during consultations. When asked about concrete learning needs with respect to *dealing with patient emotions*, most mentioned the need to be trained in dealing with difficult patient groups, such as angry, emotional, and scared patients.

*System-related strategies and challenges* (see Table 2) refer to actions undertaken by the hospital as a whole or by a separate department to facilitate information provision. Such actions included ‘teamwork’ agreements between medical specialists and clinical nurse specialists with respect to the content, level of detail, and repetition of information provision. Another frequently mentioned ‘strategy’ was the development and availability of information folders, website information, (animation) videos, and online decision aids. A few clinical nurse specialists mentioned that their department makes use of a checklist in which providers can check items discussed with the patient and a few specified sending a checklist to the patient in advance, in which patients can indicate topics they deem important to discuss in person.

*“…I think that we should work together as a team, and not expect someone else to tell certain information. We should act together and repeat certain information …” (Quote from highly experienced clinical nurse specialist)*

Time-pressure was one of the most frequently mentioned system-related challenges for effective information provision. Due to the limited time of a standard consultation, HCP found it hard to effectively organize and prioritize the information they want to offer to their patients. Developments such as an increasing demand to incorporate shared decision making during consultations and a major increase in administrative tasks that providers have to complete before, during and after consultations were perceived as making time constraints more pressing.

*“…I think that time is an important factor. Of course my goal is to explain everything to the patient, but sometimes there is insufficient time available. This forces me to postpone providing certain information to a next consultation…” (Quote from moderately experienced medical specialist)*

Finally, most medical specialists mentioned the lack of supervision or peer review regarding their communication during consultations. Although the performance of HCP in training with respect to medical procedures and medical knowledge is thoroughly checked by colleagues, this is usually not the case for the quality of provider-patient communication.

### Effective information-provision: training preferences

With respect to providers’ training preferences, four themes emerged: 1) ‘prior experience’ healthcare providers have with communication training; 2) perceived ‘facilitating factors’ and 3) perceived ‘barriers’ for developing and implementing a new (digital) communication training; and 4) ‘training format’.

#### Prior communication training experience

HCP mainly stipulated the importance of clinical experience for developing their communication skills; an opinion most frequently voiced by medical specialists. They feel they slowly and unconsciously develop better communication skills by experience. Some medical specialists mentioned that at the start of their professional careers they were insecure about their medical knowledge and were mainly occupied by ‘overthinking’ how to convey information to the patient. After gaining more and more experience this transitioned into a more ‘natural’ way of communicating, which frees up cognitive resources to listen more attentively to patients and to convey the information more effectively.

*“…I think that experience is an important factor. By seeing so many patients, you develop certain interpersonal skills, which enables you to determine what a patients deems important…” (Quote from highly experienced medical specialist)*

Some HCP had experience with communication training. This mainly entailed seniors coaching junior colleagues, providing feedback based on observed consultations. Some providers had obtained formal qualifications in training their junior colleagues. HCP frequently mentioned to have completed a course in communication skills in their undergraduate curriculum in which practicing with an actor was a main component. Some providers experienced this as a cumbersome, time-consuming, and suboptimal substitute for real life situations, which was therefore sometimes not taken seriously.

*“…It is an abstract topic which can evoke laughter and thus risks to be not taken seriously in a classroom setting. Recently I have also had to perform a roleplay tutoring session with an actor about communication, which eventually caused me to laugh and not be able to complete the task…” (Quote from highly experienced medical specialist)*

Only few HCP had experience with e-learning modules. These modules most often contained medical specific content in textual form, supported with questions, illustrations, and movie-clips. Very few brought up experience with less conventional e-learning formats, such as a serious game or interactive popups integrated in electronic health record systems.

#### Facilitating factors for implementation

One of the main facilitating factors deemed necessary by the HCP for successful implementation of a digital communication training was creating awareness of the importance of this training topic in the workplace in general, and in supervisors specifically. Although most providers brought up that they considered themselves skilled in communicating effectively with their patients and thus did not require (additional) training, they also recognized that they and/or their colleagues could probably improve certain aspects such as effective information giving through training efforts. Furthermore, some providers stipulated the importance of being able to conduct the training in their own time. Finally, there was consensus that any training should offer continuing medical education accreditation points.

*“…yes, accreditation points would motivate me to complete the training. Then there would be real value in it for me. Because there are so many things that are useful for me to do or watch, yes you have to prioritize these things and (accreditation points) would then push it to the top of my list…” (Quote from moderately experienced clinical nurse specialist)*

#### Barriers for implementation

The most frequently mentioned barrier for successful implementation of a digital communication training was lack of time, due to the aforementioned demanding nature of their jobs, but also because of being overwhelmed with other training obligations. Another limiting factor for medical specialists in particular was that they considered communication skills too difficult and abstract for training. They expressed the notion that communication effectiveness is very subjective, making it hard to evaluate an individual’s performance in a training. Moreover, interpersonal communication style differences were assumed to make it hard to develop a generic training. Also differences in organizational culture within a department were expressed as a potential barrier. Some providers found it hard to give constructive feedback to colleagues because of differences in hierarchy, and some would be reluctant to share their (recorded) consultations with peers. A few HCP warned that providers more apt in communication will be interested in training to improve their skills further, whereas the providers needing communication training most may not be interested.

*“…Of course I can hardly be improved.. Not because I’m that good at it, but due to the fact that I’m so far into my career … I’ve been doing it in a certain way for all these years, how could I possibly change something now?... But this is not a reason to not follow a training, because we’re professionals… The patient deserves a professional…” (Quote from a highly experienced medical specialist)*

#### Training format

Considering the preferred training format, most HCP expressed that a training should resemble clinical practice as closely as possible. Furthermore, reflection and feedback on their performance should be an important aspect of training. The use of video-recorded consultations, which can be reviewed by peers or a communication expert, was frequently mentioned. HCP regarded this format to be valuable provided that a safe environment is created for practicing and peer feedback. Another suggestion was to let providers reflect on their own consultations or to involve the feedback of patients in the learning module. Incorporating patient feedback in the learning experience was mentioned as an important incentive for improving communication skills. Providers also stressed the importance of pre- and post-training assessment of progress. Furthermore, video examples of role models were often mentioned as a desired learning format. Many stated that a new training should be tailored to individual learning needs. Considering a digital learning format, HCP expressed the importance of a simple, clear, and visually attractive e-learning environment in which a combination of (evidence-based) theory, modelling videos, animations, and illustrations are evenly balanced. It should be time-efficient, meaning there should not be too much text involved. A laptop or desktop computer was generally preferred over tablet or smartphone for completing an e-learning module. Finally, training was considered most effective for residents doing the training repeatedly in their curriculum. Participants also mentioned the possibility of combining digital learning with a more conventional face-to-face training (i.e. blended-learning).

*“…In an ideal world, I would want someone to observe my consultations, point out my weak spots, and offer tailored, provide guidelines how to handle certain situations.” (Quote from highly experienced medical specialist)*

## Discussion

This study is the first step in developing an innovative digital training on effective information-provision for oncological healthcare providers using a user-centred design methodology. To the best of our knowledge, no digital training tools have yet been developed for these professionals, which address the specific skill of information-provision. We assume such a digital training tool to fulfil the need for effective and easy to disseminate approaches for training communication skills of practicing HCP that benefits cancer patients. By engaging the potential users of the training tool in a systematic user-centred developmental process [39], the impact of such an intervention is more likely to be higher since health care providers learn communication best when they are motivated to learn and are engaged [21]. Based on oncological HCP’s input we conclude that to be of interest to HCP a digital training tool should facilitate individual learning needs, support feedback on competence level and improvement, and allow input from significant others such as communication experts, peers and/or patients.

### Learning needs

With respect to oncological HCP’s learning needs regarding information-provision, our main findings are that they generally struggle 1) to tailor their information to the needs of the individual patient, 2) to present such information in a structured way and 3) to deal with patients’ emotions. Thus, these topics are important to address in training.

Asking patients for their information needs and checking their level of understanding were mentioned by participants as relevant skills to tailor information. Unfortunately, however, studies show HCPs to underutilize such communication behaviors [40]. Discussions about patients’ preferences for information occurred in only 5% of hematology consultations and in less than one-third of the consultations, physicians checked patients’ understanding of presented information [41]. These findings are consistent with other studies ^e.g.^ [42]. Hence, HCP may be aware of the relevance of these skills, yet unable to apply them in actual clinical practice. Future studies should unravel the barriers HCP experience in practicing what they value.

The expressed need to learn how to tailor and structure information-giving aligns with the finding that, in a hematology setting, physicians tend to use lengthy monologues of standardized information, insufficiently tailored to patient needs, hoping to convey as much relevant information as possible in a restricted time [43]. Participants in our study seem to be conscious that this may lead to information overload, which in turn increases patients’ anxiety and hampers patients’ understanding and recall of information. Participants also realize that patients have difficulty processing cognitive information as long as their emotional concerns remain unaddressed. They know that an empathic response may be beneficial, but seem less able to clearly describe what such an empathic response might encompass. This corresponds with the results of an interview study in which oncologists indicated a preference for exploring patients’ emotions and providing empathetic statement because this would help, among other, the provision of information [44]. However, several studies show that when patients express emotions, clinicians infrequently respond empathetically [45–47] These findings clearly illustrate how HCP struggle between on the one hand knowing which skills are important for effective information-provision, yet on the other hand being unable to translate this awareness to actual practice [48]. Therefore, one of the key-features of an effective e-learning module should be to support the transfer of skills to clinical practice. Functions of an e-learning such as brief notifications or alerts that raise awareness about specific skills shortly before a consultation, or an exercise such as writing down implementation intentions [49] may help this transfer.

### Training preferences

One common theme distinctly emerged from the various participant preferences towards training: it should be neatly connected to clinical practice. This aspect of an e-learning will be more important for practicing HCP as compared to pre-clinical student learners. To align with clinical practice, participants preferred a training to be centred around video-recordings of patient consultations, either their own or of their peers. Using video recordings is a powerful tool in workplace based assessment of clinical skills [50, 51]. Reviewing these video recordings of the clinical performance allows for systematic self-reflection. Moreover, sharing video recordings of clinical performance with experts and peers enables professionals to review and exchange their evaluations and feedback. This has proven to be effective for developing critical thinking, problem solving, and self-directed learning skills through gaining new understandings, new perspectives, and new alternatives for future performance [52]. Online digital tools can be helpful in structuring this process of performance review, evaluation, reflection, and feedback [53, 54].

Participants clearly expressed that feedback from their patients would provide a strong incentive to improve their communication skills. In actual practice, patients very rarely (are invited to) provide their HCP with feedback about their communication skills. In a digital e-learning environment, however, professionals motivation to ‘satisfy’ the patient can be put to use by incorporating virtual or standardized patients who provide feedback on communication skills [29], although virtual patients can be perceived as not realistic and low levels of emotional involvement have been reported [27, 31]. Thus, in order to develop a realistic and acceptable conversation simulation technology, the learners’ perceptions and needs should be thoroughly studied and incorporated in the tool.

The wish for feedback, either from peers, a communication expert or patients, is supported by findings of a realist review by Wong and colleagues [55], who note such ‘interactivity’ to be highly valued by users of internet-based medical education. A digital tool, which allows for the uploading and annotation of videos of consultations, taking privacy issues into account, may provide a valuable resource for learning communication skills because in actual practice HCP hardly ever get or create the opportunity to attend each other’s consultation with the purpose to observe communication skills. Moreover, even professionals who consider themselves as proficient, and who are thus less inclined to engage in e-learning, may be curious to see how their colleagues perform. As a consequence, they may learn by observation of alternative and/or better ways of informing patients.

Participants also clearly expressed a wish for a training tool to be tailored to individual learning needs. Hence, an e-learning should take this into account, for example by offering a modular set of training topics which learners can select according to their own learning needs and priorities. In addition, these modules could also include role model examples, and tools to monitor skills progression, meeting these wishes expressed by the participants.

Finally, a digital training tool should, according to participants, be simple, easily accessible, and continuously available. This comes as no surprise: perceived ease of use, the degree to which a person believes that using a particular system would be free from effort, is an important aspect of the Technology Acceptance Model [56] The participants’ preferences about the design and procedures of a digital training tool fit well in the adult learning theory of self-determination, originally phrased by Deci and Ryan [57]. In general, training is most effective when guided by intrinsic motivation of the participants. Hence, as explained by self-determination theory, learners profit most from autonomy-supportive teaching approaches [58]. According to self-determination theory, intrinsic motivation is supported by three basic psychological needs: autonomy, competence (mastery), and relatedness [59]. Autonomy refers to having a sense of choice, exercising free will, which meets the need to self-select when, where and what to learn. A growing sense of competence engages motivation from within to develop expertise. This sense of competence is fed by (repeated) monitoring of actual clinical performance, possibly supported by video recordings. The third need, relatedness, to colleagues and to patients, adds a sense of purpose, aiming at meeting the standards set by experts, peers and patients, by collecting their feedback. Digital online training supporting the SDT conceptual framework may be most effective to engage trainees in learning [60].

### Strengths and limitations

We view it as our study’s strength that we managed to involve busy HCP in the development process. All participants were more or less experienced healthcare providers, who were able to reflect on their own learning experiences and learning needs, based on their actual clinical experience.

This study was limited by the relatively small number of participants (*N* = 16). Further, only a limited number of medical disciplines and medical centres was included, which may restrict the generalizability of the findings. Another potential bias might have occurred due to the fact that participation was voluntary, which possibly resulted in participation of professionals already interested in effective doctor-patient communication. Finally, findings will in part be the result of our questions.

## Conclusion

The HCP in this study show some basic awareness of important skills in effective provision of information, but still struggle with transferring this understanding systematically to their consultations in clinical practice, regardless of their level of experience. Hence, promoting transfer of skills to clinical consultations in clinical practice should be one of the key-features of an effective e-learning module. This module is probably most effective when the design enables tailoring to individual learning needs, allows feedback on individual initial competence level and improvement, and provides facilities for input from others such as peers and experts. Importantly, for successful implementation of digital education, possible barriers such as time constraints, human resources for feedback, and systematic management of continuous education need to be considered.

## Appendix

**Table 3:** Topic guide including questions

Topic 1. Learning need questions	Topic 2. Training preference questions
What is the greatest challenge encountered while informing patients about treatment?	How much time is spent on training (on and off the job)?
When thinking about informing patients specifically about treatment, what do you aim to achieve and how do you evaluate this?	Do you have experience with digital training modules?
What is the most difficult case you have encountered in daily practice with respect to this topic?	What is your view on these training modules (digital vs. classical)?
In daily practice, do you notice that given information is not recalled by patients?	What should be taken into account when developing a new (digital) training with respect to effective information-provision?
Have you observed ineffective communication skills of colleagues while informing patients?	How would you evaluate progress during the training?
Do you think that healthcare professionals should be trained in effective information-provision?	What would be facilitating factors to participate in a (digital) training module?
What would you like to learn regarding effective information-provision?	What are potential barriers to participate in a (digital) training module?

## References

[1] Oerlemans S, Husson O, Mols F (2012). Perceived information provision and satisfaction among lymphoma and multiple myeloma survivors--results from a Dutch population-based study. Ann Hematol.

[2] Hack TF, Degner LF, Parker PA (2005). SCRN communication team. The communication goals and needs of cancer patients: a review. Psychooncology.

[3] Puts MTE, Papoutsis A, Springall E, Tourangeau AE (2012). A systematic review of unmet needs of newly diagnosed older cancer patients undergoing active cancer treatment. Support Care Cancer.

[4] van Weert JCM, Jansen J, de Bruijn G-J, Noordman J, van Dulmen S, Bensing JM (2009). QUOTEchemo: a patient-centred instrument to measure quality of communication preceding chemotherapy treatment through the patient’s eyes. Eur J Cancer.

[5] Douma KFL, Koning CCE, Zandbelt LC, de Haes HCJM, Smets EMA (2012). Do patients’ information needs decrease over the course of radiotherapy?. Support Care Cancer.

[6] Wright EB, Holcombe C, Salmon P (2004). Doctors’ communication of trust, care, and respect in breast cancer: qualitative study. BMJ.

[7] Hofman M., Ryan J. L., Figueroa-Moseley C. D., Jean-Pierre P., Morrow G. R. (2007). Cancer-Related Fatigue: The Scale of the Problem. The Oncologist.

[8] Haskard Zolnierek KB, DiMatteo MR (2009). Physician communication and patient adherence to treatment. Med Care.

[9] Berrios-rivera JP, Street RL, Garcia Popa-lisseanu MG (2006). Trust in physicians and elements of the medical interaction in patients with rheumatoid arthritis and systemic lupus erythematosus. Arthritis Rheum.

[10] Case DO, Andrews JE, Johnson JD, Allard SL (2005). Avoiding versus seeking: the relationship of information seeking to avoidance, blunting, coping, dissonance, and related concepts. J Med Libr Assoc.

[11] Turner S, Maher EJ, Young T, Young J, Vaughan HG (1996). What are the information priorities for cancer patients involved in treatment decisions? An experienced surrogate study in Hodgkin’s disease. Br J Cancer.

[12] Charles C, Gafni A, Whelan T (1997). Shared decision-making in the medical encounter: what does it mean? (or it takes at least two to tango). Soc Sci Med.

[13] Dugdale David C., Epstein Ronald, Pantilat Steven Z. (1999). Time and the patient-physician relationship. Journal of General Internal Medicine.

[14] Lobb E A, Butow P N, Meiser B, Barratt A, Gaff C, Young M A, Kirk J, Suthers G K, Tucker K (2002). Tailoring communication in consultations with women from high risk breast cancer families. British Journal of Cancer.

[15] Douma KFL, Koning CCE, de Haes HCJM, Zandbelt LC, Stalpers LJA, Smets EMA (2012). Do radiation oncologists tailor information to patients needs? And, if so, does it affect patients?. Acta Oncol (Madr).

[16] Leighl N, Gattellari M, Butow P, Brown R, Tattersall MH (2001). Discussing adjuvant cancer therapy. J Clin Oncol.

[17] Pieterse AH, Jager NA, Smets EMA, Henselmans I (2013). Lay understanding of common medical terminology in oncology. Psychooncology.

[18] Politi MC, Légaré F (2010). Physicians’ reactions to uncertainty in the context of shared decision making. Patient Educ Couns.

[19] Jansen J, Butow PN, van Weert JCM (2008). Does age really matter? Recall of information presented to newly referred patients with Cancer. J Clin Oncol.

[20] Liu C, Scott KM, Lim RL, Taylor S, Calvo RA (2016). EQClinic: a platform for learning communication skills in clinical consultations. Med Educ Online.

[21] Pollak KI, Back AL, Tulsky JA (2017). Disseminating effective clinician communication techniques: engaging clinicians to want to learn how to engage patients. Patient Educ Couns.

[22] Clark RC, Mayer RE. E-Learning and the Science of Instruction : Proven Guidelines for Consumers and Designers of Multimedia Learning: John Wiley &Amp; Sons Inc; 2007.

[23] Sinclair PM, Kable A, Levett-Jones T, Booth D (2016). The effectiveness of internet-based e-learning on clinician behaviour and patient outcomes: a systematic review. Int J Nurs Stud.

[24] Feng J-Y, Chang Y-T, Chang H-Y, Scott Erdley W, Lin C-H, Chang Y-J (2013). Systematic review of effectiveness of situated e-learning on medical and nursing education. Worldviews on Evidence based Nursing.

[25] Vredenburg K, Mao J-Y, Smith PW, Carey T. A survey of user-centered design practice. In: Proceedings of the SIGCHI Conference on Human Factors in Computing Systems Changing Our World, Changing Ourselves - CHI ‘02. San Francisco: ACM Press; 2002:471.

[26] de Carvalho JMI, da Silva TS, Silveira MS. Agile and UCD Integration Based on Pre-development Usability Evaluations: An Experience Report. In: Kurosu M, ed. Human-Computer Interaction. Theory, Design, Development and Practice: 18th International Conference, HCI International 2016, Toronto, ON, Canada. Proceedings, Part I. Cham: Springer International Publishing; 2016:586–597.

[27] Ziebarth S, Kizina A, Hoppe HU, Dini L (2014). A serious game for training patient-centered medical interviews. 2014 IEEE 14th International Conference on Advanced Learning Technologies.

[28] Aper L, Reniers J, Koole S, Valcke M, Derese A (2012). Impact of three alternative consultation training formats on self-efficacy and consultation skills of medical students. Med Teacher.

[29] Courteille O, Josephson A, Larsson LO (2014). Interpersonal behaviors and socioemotional interaction of medical students in a virtual clinical encounter. BMC Med Educ.

[30] Kron FW, Fetters MD, Scerbo MW, White CB, Lypson ML, Padilla MA, Guetterman TC (2016). Using a computer simulation for teaching communication skills: a blinded multisite mixed methods randomized controlled trial. Pat Educ Couns.

[31] Quail M, Brundage SB, Spitalnick J, Allen PJ, Beilby J. Student self-reported communication skills, knowledge and confidence across standardised patient, virtual and traditional clinical learning environments. BMC Med Educ. 2016;16(73) 10.1186/s12909-016-0577-5 PMC4769506 26919838

[32] Schmitz FM, Schnabel KP, Stricker D, Fischer MR, Guttormsen S (2017). Learning communication from erroneous video-based examples: a double-blind randomised controlled trial. Pat Educ Couns.

[33] Gartmeier M, Bauer J, Fischer MR, Hoppe-Seyler T, Karsten G, Kiessling C, Prenzel M (2015). Fostering professional communication skills of future physicians and teachers: effects of e-learning with video cases and role-play. Instr Sci.

[34] Detering K, Silvester W, Corke C, Milnes S, Fullam R, Lewis V, Renton J (2014). Teaching general practitioners and doctors-in-training to discuss advance care planning: evaluation of a brief multimodality education programme. BMJ Support Palliative Care.

[35] Langenau E, Kachur E, Horber D (2014). Web-based objective structured clinical examination with remote standardized patients and skype: resident experience. Pat Educ Couns.

[36] Daetwyler CJ, Cohen DG, Gracely E, Novack DH (2010). eLearning to enhancephysician patient communication: a pilot test of “doc. Com” and “WebEncounter” in teaching bad news delivery. Med Teacher.

[37] Bekkers MJ, Simpson SA, Dunstan F, Hood K, Hare M, Evans J, Butler CC (2010). Enhancing the quality of antibiotic prescribing in primary care: qualitative evaluation of a blended learning intervention. BMC Fam Practice.

[38] Mitchell Suzanne, Heyden Robin, Heyden Neil, Schroy Paul, Andrew Stephen, Sadikova Ekaterina, Wiecha John (2011). A Pilot Study of Motivational Interviewing Training in a Virtual World. Journal of Medical Internet Research.

[39] van Gemert-Pijnen JEWC, Nijland N, van Limburg M (2011). A holistic framework to improve the uptake and impact of eHealth technologies. J Med Internet Res.

[40] Smets EMA, Hillen MA, Douma KFL, Stalpers LJA, Koning CCE, de Haes JCJM (2013). Does being informed and feeling informed affect patients’ trust in their radiation oncologist?. Pat Educ Couns.

[41] Alexander SC, Sullivan AM, Back AL, Tulsky JA, Goldman RE, Block SD, Stewart SK, Wilson-Genderson M, Lee SJ (2012). Information giving and receiving in hematological malignancy consultations. Psychooncology.

[42] Pieterse AH, Kunneman M, Engelhardt EG, Brouwer NJ, Kroep JR, Marijnen CAM, Stiggelbout AM, Smets EMA (2017). Oncologist, patient, and companion questions during pretreatment consultations about adjuvant cancer treatment: a shared decision-making perspective. Psychooncology.

[43] Chhabra KR, Pollak KI, Lee SJ, Back AL, Goldman RE, Tulsky JA (2013). Physician communication styles in initial consultations for hematological cancer. Patient Educ Couns.

[44] Visser Leonie N.C., Schepers Sanne, Tollenaar Marieke S., de Haes Hanneke C.J.M., Smets Ellen M.A. (2018). Patients’ and oncologists’ views on how oncologists may best address patients’ emotions during consultations: An interview study. Patient Education and Counseling.

[45] Pollak KI, Arnold RM, Jeffreys AS, Alexander SC, Olsen MK, Abernethy AP, Sugg Skinner C, Rodriguez KL, Tulsky JA (2007). Oncologist communication about emotion during visits with patients with advanced cancer. J Clin Oncol.

[46] Butow PN, Brown RF, Cogar S, Tattersall MH, Dunn SM (2002). Oncologists' reactions to cancer patients' verbal cues. Psychooncology.

[47] Kennifer SL, Alexander SC, Pollak KI, Jeffreys AS, Olsen MK, Rodriguez KL, Arnold RM, Tulsky JA (2009). Negative emotions in cancer care: do oncologists' responses depend on severity and type of emotion?. Patient Educ Couns.

[48] Eertwegh van den V, van Dulmen S, van Dalen J, Scherpbier AJ, van der Vleuten CP (2013). Learning in context: identifying gaps in research on the transfer of medical communication skills to the clinical workplace. Patient Educ Couns.

[49] Saddawi-Konefka D, Schumacher DJ, Baker KH, Charnin JE, Gollwitzer PM (2016). Changing Physician Behavior With Implementation Intentions: Closing the Gap Between Intentions and Actions. Acad Med.

[50] Fukkink RG, Trienekens N, Kramer L (2011). Video feedback in education and training: putting learning in the picture. Educ Psychol Rev.

[51] Weyers S, Jemi I, Karger A, Raski B, Rotthoff T, Pentzek M, Mortsiefer A. Workplace-based assessment of communication skills: A pilot project addressing feasibility, acceptance and reliability. GMS J Med Educ. 2016;33(5) Doc70 10.3205/zma001069 PMC5135413 27990466

[52] Adams CL, Nestel D, Wolf P (2006). Reflection: a critical proficiency essential to the effective development of a high competence in communication. J Vet Med Educ.

[53] Hulsman RL, van der Vloodt J (2015). Self-evaluation and peer-feedback of medical students' communication skills using a web-based video annotation system. Exploring content and specificity. Patient Educ Couns.

[54] Bergeron D, Champagne JN, Qi W, Dion M, Thériault J, Renaud JS (2018). Impact of a student-driven, virtual patient application on ObjectiveStructured clinical examination performance: observational study. J Med Internet Res.

[55] Wong G, Greenhalgh T, Pawson R (2010). Internet-based medical education: a realist review of what works, for whom and in what circumstances. BMC Med Educ.

[56] Davis FD (1989). Perceived usefulness, perceived ease of use and user acceptance of information technology. MIS Q.

[57] Deci EL, Ryan RM (1985). Intrinsic motivation and self-determination in human behaviour.

[58] Ten Cate TJ, Kusurkar RA, Williams GC (2011). How self-determination theory can assist our understanding of the teaching and learning processes in medical education. AMEE guide no. 59. Med Teach.

[59] Lyness JM, Stephen J, Lurie SJ, Ward DS, Christopher J, Mooney DJ, Lambert DR (2013). Engaging students and faculty: implications of self-determination theory for teachers and leaders in academic medicine. BMC Medical Educ.

[60] de Araujo Guerra Grangeia T, de Jorge B, Franci D, Martins Santos T, Vellutini Setubal MS, Schweller M, de Carvalho-Filho MA (2016). Cognitive Load and Self-Determination Theories Applied to E-Learning: Impact on Students' Participation and Academic Performance. PLoS One.

